# Multi-segment foot motion during single limb toe raises in healthy individuals

**DOI:** 10.1186/1757-1146-5-S1-P29

**Published:** 2012-04-10

**Authors:** Kirsten Tulchin, Wilshaw R Stevens, Kunal Singhal, Young-Hoo Kwon

**Affiliations:** 1 Movement Science Laboratory, Texas Scottish Rite Hospital for Children, Dallas, Texas, 75219, USA; 2 Department of Kinesiology, Texas Woman’s University, Denton, Texas, 76207, USA

## Background

The standing toe raise, or heel rise, requires ankle plantarflexor strength, and is often used to assess foot and ankle muscle function. There is limited research on the mechanics within the foot during this task. Houck et al., [1] found that hindfoot eversion/inversion was significantly different than controls during a bilateral toe raise task in patients with posterior tibialis tendon dysfunction. They concluded, however, that most differences in foot kinematics in this patient population occurred as an offset rather than a change in pattern of motion. However the kinematic patterns of motion during a unilateral toe raise task remain unknown. The purpose of this study was to describe the multi-segment foot kinematics during a single legged toe raise in adults without a history of foot disease.

## Materials and methods

Eighteen adults (12 males/6 females) with a mean age of 27.5 ± 5.8 yrs (range: 20.0-37.4) were instructed to perform 20 unilateral single limb toe raises, while maintaining a straight knee. Subjects were required to balance during the task on their own. The full body Plug-in-Gait model (VICON, Centennial, CO, USA) was used to assess lower extremity kinematics/kinetics, and the TSRHC kinematic multi-segment foot model [2] was applied bilaterally. Custom-written MATLAB code was used to automate the identification of each toe raise based on the position, velocity and acceleration of the heel marker on the stance limb. A single side was chosen from each subject, with a minimum of 15 completed toe raises selected for analysis for each individual. Maximal vertical excursion of the heel was determined by the displacement of the posterior calcaneus marker and was normalized relative to foot length (heel marker to metatarsal heads during the static trial.) Descriptive statistics were derived for hindfoot motion and forefoot motion (triplanar) and normalized heel excursion.

## Results

Normalized heel excursion was found to be correlated to sagittal plane hindfoot and forefoot range of motion (r^2^=0.59 and r^2^=0.49, respectively) and peak ankle power generation (r^2^=0.63). There were strong correlations in the timing of peak heel excursion and the occurrence of peak hindfoot plantarflexion (r^2^=0.89), peak forefoot plantarflexion (r^2^=0.63) and peak ankle power generation (r^2^=0.59).

There was slight hindfoot varus and forefoot eversion noted during the heel rise, however the pattern of motion in the coronal plane was not consistent across subjects or trials. Those that exhibited a hindfoot varus pattern (VAR group) had slightly higher coronal hindfoot range of motion than those that did not (8.6° vs. 5.1°, p<0.001) while those that did not have a varus pattern (NON-Var group) exhibited more hindfoot internal rotation (16.6° vs. 8.6°, p<0.001). Most differences between groups occurred at the hindfoot, with minimal differences at the forefoot. There was no significant difference in normalized heel excursion between the two groups (p=0.241). Despite instructions to keep the knee straight, mean knee flexion was approximately 13.4 ± 5.5° across all subjects, with a total range of motion of slightly over 7°.

## Conclusions

Kinematic patterns of motion within the foot during a single limb toe raises were variable among healthy young adults. Most significant differences across patterns occurred within the hindfoot, with minimal changes noted at the forefoot, which suggests that proximal motion at other joints plays a crucial role in multi-segment foot kinematics. Specifically, the effect of the control and location of the center of mass relative to the foot warrants further investigation.
